# Density Function Theory Study on the Energy and Circular Dichroism Spectrum for Methylene-Linked Triazole Diads Depending on the Substitution Position and Conformation

**DOI:** 10.3390/molecules29122931

**Published:** 2024-06-20

**Authors:** Masaki Nakahata, Akihito Hashidzume

**Affiliations:** Graduate School of Science, Osaka University, Toyonaka 560-0043, Osaka, Japan; nakahata@chem.sci.osaka-u.ac.jp

**Keywords:** DFT calculation, 1,2,3-triazole, circular dichroism

## Abstract

Since the discovery of metal-catalyzed azide–alkyne cycloadditions, 1,2,3-triazoles have been widely used as linkers for various residues. 1,2,3-Triazole is an aromatic five-membered cyclic compound consisting of three nitrogen and two carbon atoms with large dipoles that absorb UV light. In the past decade, we have been working on the synthesis of dense triazole polymers possessing many 1,2,3-triazole residues linked through a carbon atom in their backbone as a new type of functional polymer. Recently, we reported that stereoregular dense triazole uniform oligomers exhibit a circular dichroism signal based on the chiral arrangement of two neighboring 1,2,3-triazole residues. In this study, to investigate the chiral conformation of two neighboring 1,2,3-triazole residues in stereoregular dense triazole uniform oligomers, density functional theory (DFT) calculations were performed using 1,2,3-triazole diads with different substitution positions and conformations as model compounds and compared with our previous results.

## 1. Introduction

The development of synthetic (macro)molecules with structures and functions like those of peptides and proteins is a major challenge in organic chemistry and macromolecular science. In particular, an amide bond alternative to connect units is an important aspect that underlies the design of such molecules. 1,2,3-Triazoles, which are formed by azide–alkyne cycloaddition (AAC), have been extensively studied as amide bond alternatives [1]. Thermal AAC without any catalyst, called Huisgen cycloaddition [2], is known to yield a mixture of 1,4- and 1,5-disubstituted 1,2,3-triazoles. On the other hand, metal-catalyzed AAC reactions have the potential to yield selective structural isomers. AAC catalyzed by a copper(I) compound (copper(I)-catalyzed AAC, CuAAC) selectively produces 1,4-disubstituted 1,2,3-triazoles, whereas that catalyzed by a ruthenium complex with a bulky ligand, e.g., pentamethylcyclopentadienyl, (ruthenium-catalyzed AAC, RuAAC), preferentially produces 1,5-disubstituted 1,2,3-triazoles [3,4,5]. These reactions have been extensively used to connect building blocks due to their high selectivity and versatility [6,7,8,9,10,11,12,13,14]. Hashidzume et al. [15,16,17] have reported on the synthesis of 4-azido-5-hexynoate (AH) derivatives, in which azide and alkyne groups are linked through methylene or methine (C1) linkers, and the synthesis of oligomers and polymers of AH derivatives by CuAAC polymerization. The polymers of AH derivatives have structural similarity to proteins in terms of the C1 linkers linking planar structures (1,2,3-triazole/amide bonds).

The molecular conformation of polypeptides and proteins is closely related to their properties, i.e., some polypeptide chains fold into a specific structure through noncovalent bonds in aqueous media, which exert biological functions. The folded structure of polypeptide chains is characterized by the dihedral angles (*θ* and *ψ*) between adjacent amide planes of amino acid residues [18]. Here, *θ* and *ψ* are defined as the dihedral angles in the chains C′ (carbonyl carbon)–N (amide nitrogen)–C_α_ (amino acid carbon)–C′ and N–C_α_–C′–N, respectively. Typical secondary structures of proteins, i.e., α-helix and β-sheet, are known to have specific *θ* and *ψ* values in the Ramachandran plot [19]. This plot helps not only in the classification of protein structural motifs [20] but also in the design of de novo proteins [21]. On the other hand, circular dichroism (CD) spectroscopy is one of the most frequently used experimental methods for characterizing the steric conformation of chromophores. The point here is that the CD spectra provide information about the steric conformation of moieties having dipole moments, e.g., amide bonds in folded proteins [22]. This method is commonly used to predict the tertiary structure of proteins from the characteristic CD signals ascribable to their secondary structures [23].

In this study, we systematically investigate 1,2,3-triazole diads linked through a methylene linker and focus on properties such as the formation energy and CD spectra, depending on the conformation of the triazole moieties. To the best of our knowledge, there have been a few publications in which conformations and properties of similar triazole-containing molecules were discussed and most used experimental techniques [24,25,26]. However, a comprehensive study on this topic using computational methods, e.g., density functional theory (DFT) and time-dependent DFT (TDDFT) calculations, has not yet been reported. Here, we designed four structural isomers of methylene-linked diads of 1,4- and/or 1,5-disubstituted 1,2,3-triazoles as model molecules. The values of the formation energy and simulated CD spectra for the 1,2,3-triazole diads, depending on the dihedral angles of two 1,2,3-triazole rings, were investigated using DFT and TDDFT calculations and compared with the four structural isomers. Finally, the calculated values were compared with our previous experimental results [17].

## 2. Results and Discussion

### 2.1. Model Molecules and Definition of Dihedral Angles

**Figure 1:**
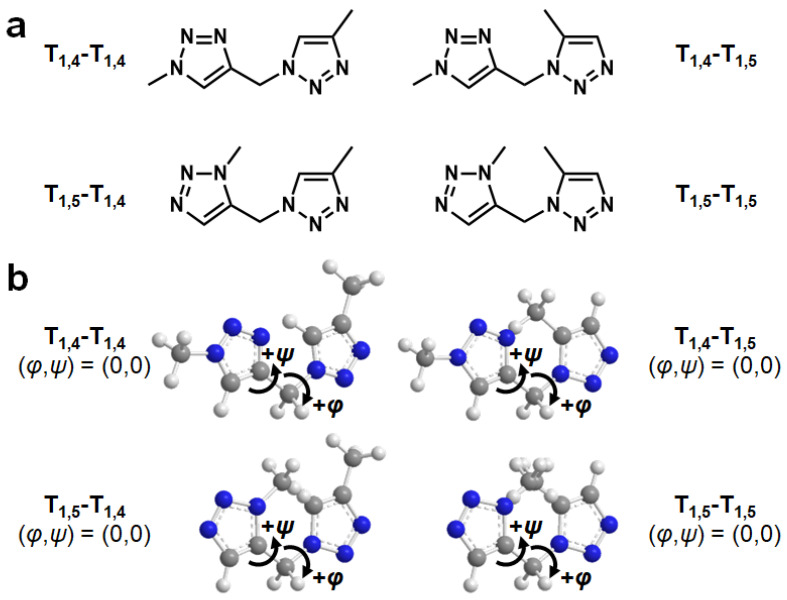
(**a**) Chemical structures of the model compounds (T_1,4_-T_1,4_, T_1,4_-T_1,5_, T_1,5_-T_1,4_, T_1,5_-T_1,5_). (**b**) Definition of dihedral angles *φ* and *ψ* for T_1,4_-T_1,4_, T_1,4_-T_1,5_, T_1,5_-T_1,4_, and T_1,5_-T_1,5_. White, gray, and blue atoms indicate hydrogen, carbon, and nitrogen, respectively. Dashed lines indicate conjugated π bonds.

Figure 1a depicts four structural isomers as model molecules for disubstituted 1,2,3-triazole diads. The hydrogen atoms on 1,2,3-triazole nitrogen (left) and those on 1,2,3-triazole carbon (right) are each substituted with a methyl group. We refer to the structural isomers shown here as T_1,4_-T_1,4_, T_1,4_-T_1,5_, T_1,5_-T_1,4_, and T_1,5_-T_1,5_.

Figure 1b illustrates the definitions of the dihedral angles *φ* and *ψ* in the four model structures, where *φ* denotes the dihedral angle between C (triazole)–C (methylene linker)–N (triazole)–C (triazole) and *ψ* denotes that between N (triazole)–C (methylene linker)–C (triazole)–N (triazole). The conformers shown in Figure 1b are defined as (*φ*,*ψ*) = (0,0), and the positive and negative directions of *φ* and *ψ* are defined as shown in Figure 1b. The dihedral angles (*φ* and *ψ*) were set to 0°, ±30°, ±60°, ±90°, ±120°, ±150°, and 180° using three-dimensional molecular modeling software (PerkinElmer Chem3D Ultra (version 21.0.0.28)) to prepare 144 conformers for each structural isomer, and DFT calculations for these conformers were performed using Gaussian 09. Here, we used B3LYP/6-31+G(d) as basis functions, taking into account the contributions of the polarization and dispersion functions with the minimum calculation cost.

### 2.2. Calculation of the Ground State: The Formation Energy

**Figure 2:**
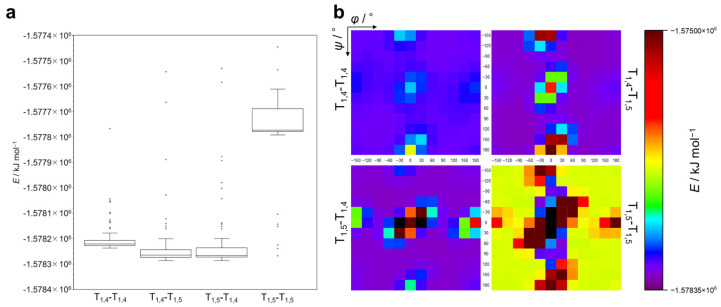
(**a**) Box-and-whisker plot of formation energy for T_1,4_-T_1,4_, T_1,4_-T_1,5_, T_1,5_-T_1,4_, and T_1,5_-T_1,5_. (**b**) Formation energy map as a function of *φ* and *ψ* for T_1,4_-T_1,4_, T_1,4_-T_1,5_, T_1,5_-T_1,4_, and T_1,5_-T_1,5_. The color of each dot corresponds to the *E* value in the color bar. Black dot: no data (calculation unsuccessful).

First, calculations for the ground state were performed for T_1,4_-T_1,4_, T_1,4_-T_1,5_, T_1,5_-T_1,4_, and T_1,5_-T_1,5_ to obtain the values for the formation energy (*E*) for each conformer. Tables S1–S4 (Supplementary Materials) present the *E* values (in kJ mol^–1^) for each structural isomer. Figure 2a displays the *E* values as a box-and-whisker diagram, with outlier points plotted. Figure 2b shows the *E* values for conformers of varying *φ* and *ψ* values for four structural isomers in a heatmap as a function of *φ* (X-axis) and *ψ* (Y-axis). Each dot in the heatmap is colored according to the *E* value indicated in the color bar, and a black dot means that the calculation for the corresponding conformer was unsuccessful, presumably due to an extremely large steric hindrance of the methyl groups (see Figure 1b (T_1,5_-T_1,5_, (*φ*,*ψ*) = (0,0)) for an example). The following should be noted here. (1) For T_1,4_-T_1,4_, T_1,4_-T_1,5_, and T_1,5_-T_1,4_, the *E* values of most conformers are within a similar range, whereas T_1,5_-T_1,5_ exhibits higher *E* values compared to other structural isomers. (2) In Figure 2b, energy minimum regions similar to those in the Ramachandran plot exist for all structural isomers (see also Figure S1). Intriguingly, T_1,5_-T_1,5_ shows narrow and deep energy minimum regions, while T_1,4_-T_1,4_, T_1,4_-T_1,5_, and T_1,5_-T_1,4_ exhibit relatively wide and shallow energy minimum regions. (3) The *E* heatmaps seem to show symmetric patterns corresponding to the positive and negative values of *φ* and *ψ*, with the most stable combinations of *φ* and *ψ* for each structural isomer as follows: T_1,4_-T_1,4_: (*φ*,*ψ*) = (90,−120), (−90,120); T_1,4_-T_1,5_: (*φ*,*ψ*) = (90,−90), (−90,90); T_1,5_-T_1,4_: (*φ*,*ψ*) = (60,60), (−60,−60); and T_1,5_-T_1,5_: (*φ*,*ψ*) = (30,60), (−30,−60). (4) T_1,5_-T_1,5_ has the widest region where calculations were unsuccessful. These observations indicate that T_1,5_-T_1,5_ mainly takes relatively unstable conformations compared to other structural isomers, presumably because of the steric hindrance between methyl substituents. As can be seen in Figure S2 in the Supplementary Materials, there is a CH–π interaction between the methyl and triazole moieties in the most stable conformers of the T_1,5_-T_1,4_ diad with (60,60) and the T_1,5_-T_1,5_ diad with (30,60). It is thus likely that the CH–π interaction makes these conformers stable.

### 2.3. Simulation and Classification of CD Spectra

**Figure 3:**
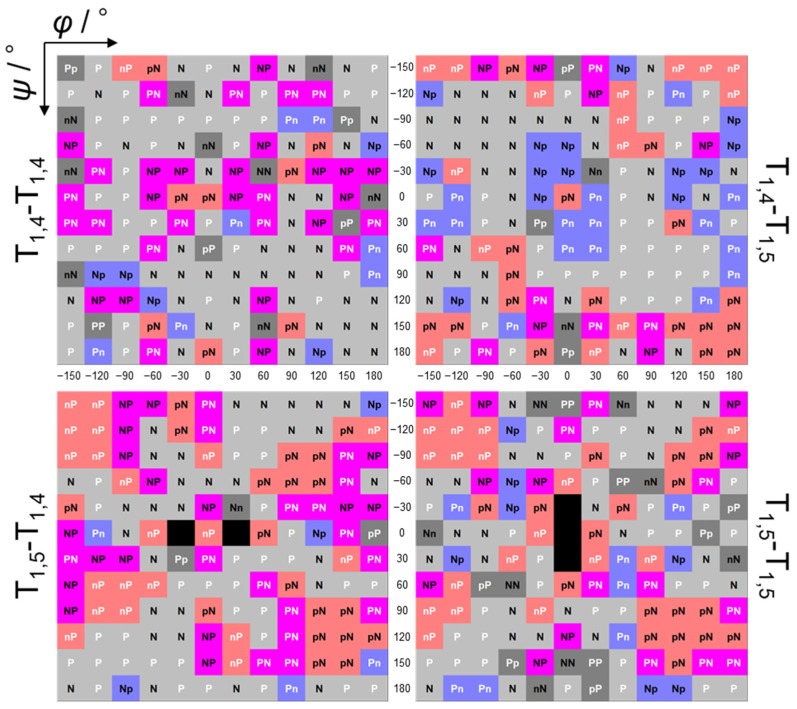
Classification of simulated CD spectra based on *φ* and *ψ* for T_1,4_-T_1,4_, T_1,4_-T_1,5_, T_1,5_-T_1,4_, and T_1,5_-T_1,5_.

Next, calculations were performed for the singlet excited state of all structural isomers. The calculations produce simulated CD spectra, which contain a variety of spectra including simple Cotton-effect types, exciton-coupling types, and others. These spectra were classified as shown in Figure S3 and as follows: a letter was assigned to each peak in the spectrum in order, from longest to shortest wavelength (moving from left to right on the X-axis of each panel in Figure S3) according to the peak intensity (uppercase: larger peak, lowercase: smaller peak) and positive/negative value (P/p: positive, N/n: negative). A peak with an intensity greater than half of the largest peak was regarded as another large peak (P or N), while a peak with an intensity smaller than half of the largest peak was considered a small peak (p or n). Figure 3 shows the classification of CD spectra for conformers differing by 30° in *φ* and *ψ* for each structural isomer in a color map. The following should be noted. (1) From the color map of the CD spectra for all structural isomers, the shapes of the CD spectra depending on *φ* and *ψ* are not entirely random, and there seem to be regions of *φ* and *ψ* that produce similar CD spectra (see also Figure S4). (2) The color map of the CD spectra shows symmetric patterns corresponding to the positive and negative values of *φ* and *ψ*. (3) The energetically stable regions (see Figure S1) do not necessarily correspond to the trends observed in the CD spectra. As discussed below, these simulated CD spectra can provide fundamental data for predicting conformations based on the CD spectra of triazole oligomers with unknown conformations.

### 2.4. Simulated CD Spectra Considering Boltzmann Distribution

**Figure 4:**
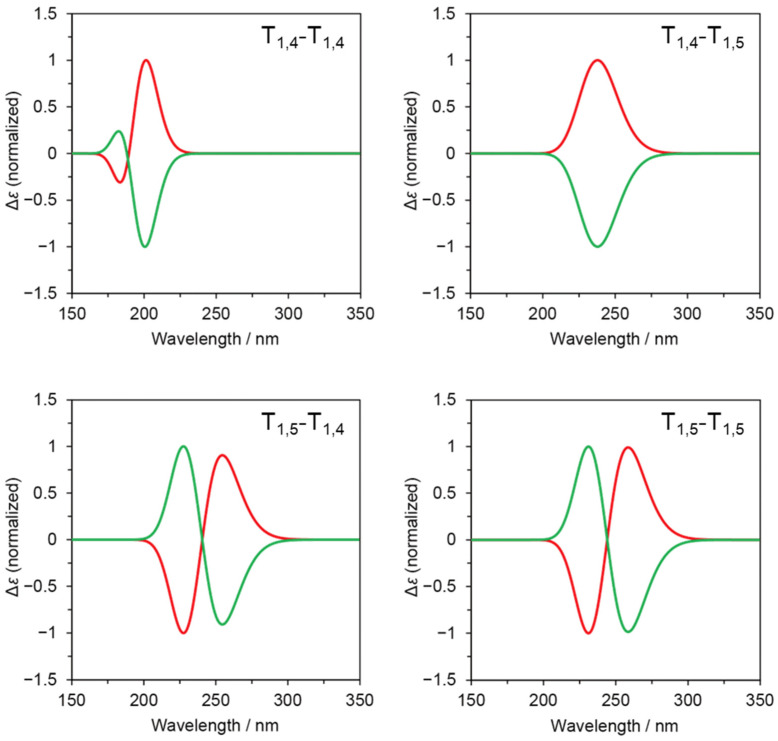
Simulated CD spectra for sum of stable conformers according to Boltzmann distribution for T_1,4_-T_1,4_, T_1,4_-T_1,5_, T_1,5_-T_1,4_, and T_1,5_-T_1,5_.

Furthermore, we combined the formation energy (*E*) and simulated CD spectra to simulate CD spectra that consider the contribution of energetically stable conformers. According to the Boltzmann distribution, the molar ratio (*N*/*N*_0_) of a conformer that is unstable by an energy difference of Δ*E* (>0) from the most stable conformer is given by *N*/*N*_0_ = exp(−Δ*E*/*RT*), where *R* and *T* denote the gas constant and absolute temperature. Tables S5-S8 show *N*/*N*_0_ values for five pairs ((*φ*,*ψ*) and (−*φ*,−*ψ*)) of energetically stable conformers of T_1,4_-T_1,4_, T_1,4_-T_1,5_, T_1,5_-T_1,4_, and T_1,5_-T_1,5_. Figure 4 presents simulated CD spectra for these pairs based on the Boltzmann distribution. The sum of simulated CD spectra are mirror images of each other, showing a simple Cotton-effect type (similar to “P” and “N” in Figure S3) for T_1,4_-T_1,5_, an exciton-coupling type (similar to “PN” and “NP” in Figure S3) for T_1,5_-T_1,4_ and T_1,5_-T_1,5_, and an in-between type (similar to “Pn” and “Np” in Figure S3) for T_1,4_-T_1,4_. These observations are influenced by the relative configuration of the 1,2,3-triazole rings in the energetically stable conformers.

### 2.5. Comparison of the Simulated CD Spectra with the Experimental Data

**Figure 5:**
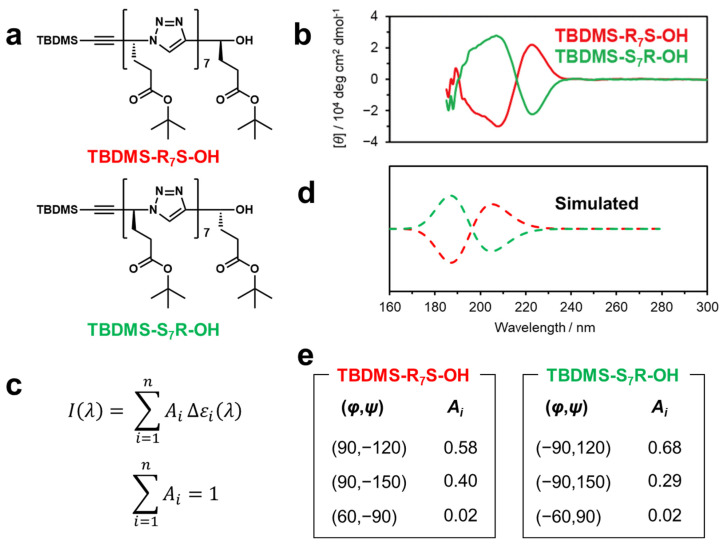
Comparison of simulated CD spectra with experimental results in the previous report [17]. (**a**) Chemical structures of TBDMS-R_7_S-OH and TBDMS-S_7_R-OH. (**b**) CD spectra of TBDMS-R_7_S-OH (red) and TBDMS-S_7_R-OH (green) in acetonitrile (original data from [17]). (**c**) Equation for fitting CD spectra. (**d**) Simulated CD spectra. (**e**) Fitting parameters.

Finally, we reproduced the experimentally observed CD spectra for stereoregular uniform oligomers possessing a dense 1,2,3-triazole backbone from our previous work using the simulated CD spectra obtained through the proposed calculations. Figure 5a shows the chemical structures of the stereoregular octamers (TBDMS-R_7_S-OH and TBDMS-S_7_R-OH) synthesized and measured for CD spectra in solution in our previous study [17]. The carbon atoms linking the 1,4-disubstituted 1,2,3-triazoles were modified with *t*-butyl propionate as side chains, and the stereochemistry of the carbon atoms connecting the triazole groups was regulated as either an *R* or *S* configuration. The experimentally obtained CD spectra of TBDMS-R_7_S-OH and TBDMS-S_7_R-OH in acetonitrile are shown in the upper panel of Figure 5b, demonstrating that they show mirror-image spectra. Here, using the simulated CD spectra of three stable conformers of T_1,4_-T_1,4_, i.e., T_1,4_-T_1,4_ (±90,∓120), (±60,∓150), and (±90,∓150) (see Table S5), we simulated the CD spectra by linearly combining these spectra with an arbitrary coefficient, *A*_i_ (Figure 5c). Figure 5d shows the simulated CD spectra obtained by summing the CD spectra weighted with the respective coefficients as fitting parameters, which are shown in Figure 5e. Notably, the spectral shapes of the experimentally obtained CD spectra are similar to those of the spectra simulated using the *A_i_* values, which indicate the larger contributions of stable conformers. It should also be noted that the *A_i_* values are larger for more stable conformers (see Table S5). The current data do not accurately reproduce the wavelengths, which is likely due to differences in the chemical structure between the experimentally obtained compounds and the models, e.g., the structures of the side chains and the stereochemistry of chiral carbon atoms.

## 3. Materials and Methods

### Calculation

The initial molecular structures were constructed using the Chem3D software (version 21.0.0.28) installed on a Windows 10 computer (Redmond, DC, USA). Density functional theory (DFT) and time-dependent DFT (TDDFT) calculations were carried out using the Gaussian 09 program [27]. In the calculations of the total energies and CD spectra for model triazole diads, DFT and TDDFT with the B3LYP functional were used, respectively, and the 6-31+G(d) basis sets were applied for the hydrogen, carbon, and nitrogen atoms. TDDFT calculations were performed for up to three excited states with lower levels of excitation energy.

## 4. Conclusions

In this study, we aimed to gain insights through computational methods on how the conformation of adjacent 1,2,3-triazoles in dense triazole oligomers affects their stability and CD spectra. To achieve this, we designed model molecules of 1,2,3-triazole diads and performed DFT calculations. The four designed structural isomers exhibited different stabilities based on their structures and triazole conformations; T_1,5_-T_1,5_ is less stable than the other structural isomers. We predicted the CD spectra of these structural isomers based on the relationship between the simulated CD spectra and the energetic stability of their conformers. In addition, we compared the simulated CD spectra of energetically stable conformers with those experimentally obtained for the dense triazole oligomers. These findings suggest that our approach shows promise as a powerful tool for predicting the structures of dense triazole oligomers and polymers, much like the Ramachandran plot for proteins. For example, potential applications of this work include simulation tools that estimate the conformation of highly dense triazole polymers from experimentally observed CD spectra and tools for de novo designs of dense triazole oligomers and polymers with specific folded conformations in solution.

## Data Availability

All data are contained within the article.

## Supplementary Materials

The following supporting information can be downloaded at https://www.mdpi.com/article/10.3390/molecules29122931/s1: Tables S1: Formation energy for structural isomers; Figure S1: Formation energy map; Figure S2: Most stable conformers for T_1,5_-T_1,4_ and T_1,5_-T_1,5_; Figures S3 and S4: Classification of CD spectra; Tables S5–S8: Boltzmann distribution.
