# Supplementary material for: Density Function Theory Study on the Energy and Circular Dichroism Spectrum for Methylene-Linked Triazole Diads Depending on the Substitution Position and Conformation
Source: Molecules. 2024 Jun 20;29(12):2931. doi: 10.3390/molecules29122931 (PMC11206612; doi:10.3390/molecules29122931)
Supplement: Supplementary file 1 [file molecules-29-02931-s001.zip › molecules-3048467-supplementary.pdf]

Supporting Materials

Density Function Theory Study on the Energy and Circular Dichroism Spectrum for Methylene-Linked Triazole Diads Depending on the Substitution Position and Conformation

Masaki Nakahata and Akihito Hashidzume *

Graduate School of Science, Osaka University, Toyonaka 560-0043, Osaka, Japan; nakahata@chem.sci.osaka-u.ac.jp
* Correspondence: hashidzume@chem.sci.osaka-u.ac.jp

Table S1. Formation energy *E* (kJ mol^{-1}) for $T_{1,4}$-$T_{1,4}$ with φ and ψ of 0°, ±30°, ±60°, ±90°, ±120°, ±150°, and 180°.

ψ \ φ (°)	−150	−120	−90	−60	−30	0	30	60	90	120	150	180
−150	−1578227.04628895	−1578227.62403022	−1578227.18194854	−1578206.60716527	−1578051.71812947	−1578096.35756310	−1578205.65959607	−1578231.93381473	−1578236.44216580	−1578235.54860313	−1578226.86027228	−1578222.61397236
−120	−1578226.06545466	−1578224.35284101	−1578223.58821664	−1578210.83997012	−1578183.03566257	−1578197.76143569	−1578223.78491911	−1578235.64296360	−1578237.44694465	−1578235.74766854	−1578231.48569439	−1578227.84620003
−90	−1578221.26690707	−1578219.59328210	−1578221.79352987	−1578222.48316995	−1578216.83015339	−1578215.48340316	−1578226.53450024	−1578236.51121645	−1578235.30430036	−1578231.68927566	−1578228.62295421	−1578225.19087435
−60	−1578207.24019958	−1578210.74894404	−1578219.31206480	−1578224.32312035	−1578219.56828734	−1578204.18873846	−1578207.75106937	−1578229.00735366	−1578230.73459135	−1578226.47676549	−1578221.86670255	−1578213.99960184
−30	−1578180.67588942	−1578201.75700036	−1578216.72970176	−1578220.53197711	−1578203.12764264	−1578137.31951139	−1578152.89098432	−1578214.96518197	−1578222.84911214	−1578217.64513485	−1578205.09335448	−1578183.26389729
0	−1578179.22144119	−1578206.73153521	−1578217.94163256	−1578214.80345117	−1578159.91212267	−1578056.42292045	−1578155.72303240	−1578214.45478478	−1578218.02604238	−1578206.24957218	−1578178.60531511	−1578153.97061618
30	−1578206.18230687	−1578218.56931085	−1578223.23519191	−1578216.00046914	−1578155.97809973	−1578135.20650899	−1578202.09776400	−1578221.00695632	−1578217.25645583	−1578201.63940421	−1578180.28324590	−1578183.48543698
60	−1578223.04907022	−1578227.44308076	−1578231.01756774	−1578229.39815934	−1578208.66891792	−1578203.92823635	−1578219.66642853	−1578224.93299774	−1578219.79163863	−1578210.58416766	−1578206.76742579	−1578214.33918401
90	−1578229.74399020	−1578232.63752750	−1578235.49948002	−1578236.61857315	−1578227.13912663	−1578215.93057833	−1578216.95901293	−1578222.89083133	−1578222.09548862	−1578219.33994760	−1578220.77785519	−1578225.50204861
120	−1578232.42622726	−1578236.54450779	−1578237.60589242	−1578235.66139461	−1578224.40871165	−1578198.63065998	−1578182.58767351	−1578210.08036046	−1578223.68696170	−1578223.97038443	−1578225.42525274	−1578227.98873843
150	−1578227.44709778	−1578236.10058825	−1578236.35145478	−1578231.79602849	−1578207.01044208	−1578101.06876030	−1578043.25464643	−1578204.66471536	−1578227.13857527	−1578227.04437233	−1578226.15044210	−1578222.39211761
180	−1578222.21980605	−1578231.29293018	−1578231.40406760	−1578222.82519383	−1578132.65247510	−1577766.60366355	−1578125.58287002	−1578222.36124173	−1578231.52646840	−1578230.70431933	−1578221.37526146	−1578209.14421218

Table S2. Formation energy *E* (kJ mol^{-1}) for $T_{1,4}$-$T_{1,5}$ with φ and ψ of 0°, ±30°, ±60°, ±90°, ±120°, ±150°, and 180°.

ψ \ φ (°)	−150	−120	−90	−60	−30	0	30	60	90	120	150	180
−150	−1578272.06600000	−1578274.69150000	−1578272.06600000	−1578122.41250000	−1577198.23650000	−1577219.24050000	−1578206.42850000	−1578279.94250000	−1578285.19350000	−1578282.56800000	−1578269.44050000	−1578264.18950000
−120	−1578272.06600000	−1578272.06600000	−1578266.81500000	−1578140.79100000	−1578027.89450000	−1578161.79500000	−1578264.18950000	−1578285.19350000	−1578285.19350000	−1578282.56800000	−1578277.31700000	−1578274.69150000
−90	−1578269.44050000	−1578269.44050000	−1578269.44050000	−1578261.56400000	−1578240.56000000	−1578245.81100000	−1578258.93850000	−1578282.56800000	−1578285.19350000	−1578279.94250000	−1578277.31700000	−1578272.06600000
−60	−1578261.56400000	−1578264.18950000	−1578272.06600000	−1578274.69150000	−1578264.18950000	−1578201.17750000	−1578151.29300000	−1578258.93850000	−1578282.56800000	−1578277.31700000	−1578272.06600000	−1578264.18950000
−30	−1578243.18550000	−1578256.31300000	−1578266.81500000	−1578272.06600000	−1578224.80700000	−1577886.11750000	−1577888.74300000	−1578253.68750000	−1578274.69150000	−1578266.81500000	−1578258.93850000	−1578245.81100000
0	−1578243.18550000	−1578258.93850000	−1578266.81500000	−1578266.81500000	−1578025.26900000	−1577542.17700000	−1578027.89450000	−1578266.81500000	−1578266.81500000	−1578258.93850000	−1578243.18550000	−1578227.43250000
30	−1578258.93850000	−1578266.81500000	−1578274.69150000	−1578251.06200000	−1577888.74300000	−1577886.11750000	−1578224.80700000	−1578272.06600000	−1578266.81500000	−1578256.31300000	−1578243.18550000	−1578245.81100000
60	−1578272.06600000	−1578277.31700000	−1578282.56800000	−1578258.93850000	−1578151.29300000	−1578201.17750000	−1578264.18950000	−1578274.69150000	−1578272.06600000	−1578264.18950000	−1578261.56400000	−1578264.18950000
90	−1578277.31700000	−1578279.94250000	−1578285.19350000	−1578282.56800000	−1578258.93850000	−1578245.81100000	−1578240.56000000	−1578261.56400000	−1578269.44050000	−1578269.44050000	−1578269.44050000	−1578272.06600000
120	−1578277.31700000	−1578282.56800000	−1578285.19350000	−1578285.19350000	−1578264.18950000	−1578161.79500000	−1578027.89450000	−1578140.79100000	−1578266.81500000	−1578272.06600000	−1578272.06600000	−1578274.69150000
150	−1578269.44050000	−1578282.56800000	−1578285.19350000	−1578279.94250000	−1578206.42850000	−1577219.24050000	−1577198.23650000	−1578122.41250000	−1578272.06600000	−1578274.69150000	−1578272.06600000	−1578264.18950000
180	−1578264.18950000	−1578279.94250000	−1578279.94250000	−1578261.56400000	−1577662.95000000	−1575819.84900000	−1577662.95000000	−1578261.56400000	−1578279.94250000	−1578279.94250000	−1578264.18950000	−1578243.18550000

Table S3. Formation energy E (kJ mol^{-1}) for $T_{1,5}$-$T_{1,4}$ with φ and ψ of 0°, ±30°, ±60°, ±90°, ±120°, ±150°, and 180°.

$\psi \setminus \varphi$ (°)	−150	−120	−90	−60	−30	0	30	60	90	120	150	180
−150	−1578262.76826434	−1578268.93748046	−1578270.45575460	−1578254.41387083	−1578160.60900914	−1578205.28401830	−1578264.14972368	−1578275.23970440	−1578274.62145166	−1578268.66385084	−1578252.58397610	−1578250.26923028
−120	−1578266.14877938	−1578269.17671601	−1578270.44246956	−1578263.93175466	−1578255.92334955	−1578265.02587928	−1578272.51706090	−1578274.04008720	−1578272.18144324	−1578268.53840446	−1578263.89000922	−1578263.18122924
−90	−1578274.03853815	−1578278.02499233	−1578280.32133339	−1578280.46591968	−1578278.52780183	−1578276.38820312	−1578274.88741481	−1578270.73873098	−1578272.35406986	−1578271.71470810	−1578270.34474846	−1578270.92146579
−60	−1578261.43107093	−1578262.35902766	−1578282.48681954	−1578286.64847334	−1578283.21557957	−1578267.69241584	−1578143.27545814	−1578138.75114403	−1578263.51172717	−1578274.37875044	−1578275.84057633	−1578275.21922550
−30	−1577892.43584452	−1578150.72778236	−1578261.94934463	−1578271.87951073	−1578212.73348072	−1577583.67470782	−1576999.29572248	−1578001.45266942	−1578239.77452917	−1578266.63399803	−1578235.15441056	−1578042.54801936
0	−1577876.20794408	−1578200.08547578	−1578248.61130579	−1578152.48371676	N.D.	−1575158.01566427	N.D.	−1578152.48828513	−1578248.60768260	−1578200.05767174	−1577876.12300915	−1577529.67415896
30	−1578235.17378675	−1578266.63549456	−1578239.78314081	−1578001.44035583	−1576999.31501991	−1577583.53857565	−1578212.73650004	−1578271.87444352	−1578261.94808440	−1578150.74282648	−1577892.53931547	−1578042.62686313
60	−1578275.84496091	−1578274.38463156	−1578263.52595738	−1578138.76920747	−1578143.27784734	−1578267.70661980	−1578283.21720738	−1578286.64716059	−1578282.49918565	−1578262.39483948	−1578261.47061097	−1578275.23610746
90	−1578270.35083962	−1578271.72342476	−1578272.36843135	−1578270.75973498	−1578274.90991535	−1578276.40167194	−1578278.53179259	−1578280.46951661	−1578280.33664006	−1578278.04759788	−1578274.06025103	−1578270.93446201
120	−1578263.88730495	−1578268.53850947	−1578272.18795447	−1578274.05586645	−1578272.53152740	−1578265.03034263	−1578255.92056651	−1578263.92823650	−1578270.44685415	−1578269.18632534	−1578266.15896632	−1578263.18435358
150	−1578252.57360537	−1578268.65631566	−1578274.62058524	−1578275.24823728	−1578264.15618240	−1578205.28063140	−1578160.61173966	−1578254.40124217	−1578270.44984722	−1578268.93721790	−1578262.76897323	−1578250.26345418
180	−1578249.07368260	−1578269.00230405	−1578274.01335961	−1578268.82518782	−1578205.18122997	−1577978.91254188	−1578205.17458746	−1578268.81276921	−1578274.01020900	−1578269.00516584	−1578249.07775212	−1578224.43759215

Table S4. Formation energy E (kJ mol^{-1}) for $T_{1,5}$-$T_{1,5}$ with φ and ψ of 0°, ±30°, ±60°, ±90°, ±120°, ±150°, and 180°.

$\psi \setminus \varphi$ (°)	−150	−120	−90	−60	−30	0	30	60	90	120	150	180
−150	−1577785.27036468	−1577779.80286595	−1577771.31761752	−1577624.92412211	−1576873.22152292	−1577211.11977599	−1577722.85143887	−1577774.66838564	−1577776.92584305	−1577779.65652058	−1577775.09912517	−1577778.59531974
−120	−1577771.87041654	−1577775.55895524	−1577768.93095926	−1577640.24640883	−1577535.06764484	−1578152.64077417	−1577775.63036884	−1577774.05236457	−1577774.15895987	−1577773.45466950	−1577771.87957954	−1577772.44366822
−90	−1577775.12748057	−1577780.93435143	−1577782.04777347	−1577770.75345008	−1577758.90913695	−1578239.47847778	−1578224.88369085	−1577723.73807023	−1577751.55508519	−1577774.80693327	−1577773.56394281	−1577773.00875458
−60	−1577772.03403771	−1577775.84923052	−1577790.01445940	−1577792.12827570	−1578267.85868876	−1578103.41472332	−1576693.25323853	−1576309.39647438	−1577445.08133661	−1577775.54427869	−1577779.78574769	−1577777.87296592
−30	−1577660.76484886	−1577688.61709804	−1577772.69295944	−1577769.00549720	−1578143.43012635	N.D.	−1566216.68165236	−1575968.32837640	−1577702.21156947	−1577774.26211577	−1577761.93077239	−1577663.05932582
0	−1577397.20930861	−1577708.22133897	−1577746.04437073	−1577610.56137686	−1575382.52101404	N.D.	−1575383.63047158	−1577610.65011877	−1577746.04636612	−1577708.20826398	−1577396.99992499	−1575716.01026496
30	−1577761.92294840	−1577774.24234576	−1577702.17670283	−1575967.56138908	−1566218.10385946	N.D.	−1578143.50400792	−1577769.00578601	−1577772.68849609	−1577688.64309049	−1577660.84944247	−1577663.09949597
60	−1577779.76697537	−1577775.53120370	−1577445.36525818	−1576309.57857906	−1576694.95548145	−1578103.54329405	−1578267.85606326	−1577792.12853825	−1577790.01992043	−1577775.86086148	−1577772.04766405	−1577777.87262460
90	−1577773.55488484	−1577774.79824287	−1577751.57299111	−1577723.79801039	−1578224.94315843	−1578239.48065695	−1578238.87626685	−1577770.76426714	−1577782.06581066	−1577780.95695698	−1577775.14425751	−1577773.01697239
120	−1577771.87041654	−1577773.44075435	−1577774.15134592	−1577774.05882330	−1577775.62863601	−1578152.63996027	−1577535.08276772	−1577640.25769848	−1577768.93702416	−1577775.58003801	−1577774.94506083	−1577772.45196480
150	−1577775.09539696	−1577779.65216225	−1577776.91484221	−1577774.66534006	−1577722.85356553	−1577212.27893424	−1576873.28639903	−1577624.79859695	−1577771.32357741	−1577779.82137572	−1577785.29094860	−1577778.60571672
180	−1577782.01463966	−1577783.62879706	−1577775.72591079	−1577758.05984021	−1577258.77609290	−1575806.03052091	−1577258.31187825	−1577758.04933821	−1577775.72822122	−1577783.64455006	−1577782.02868609	−1577765.62235667

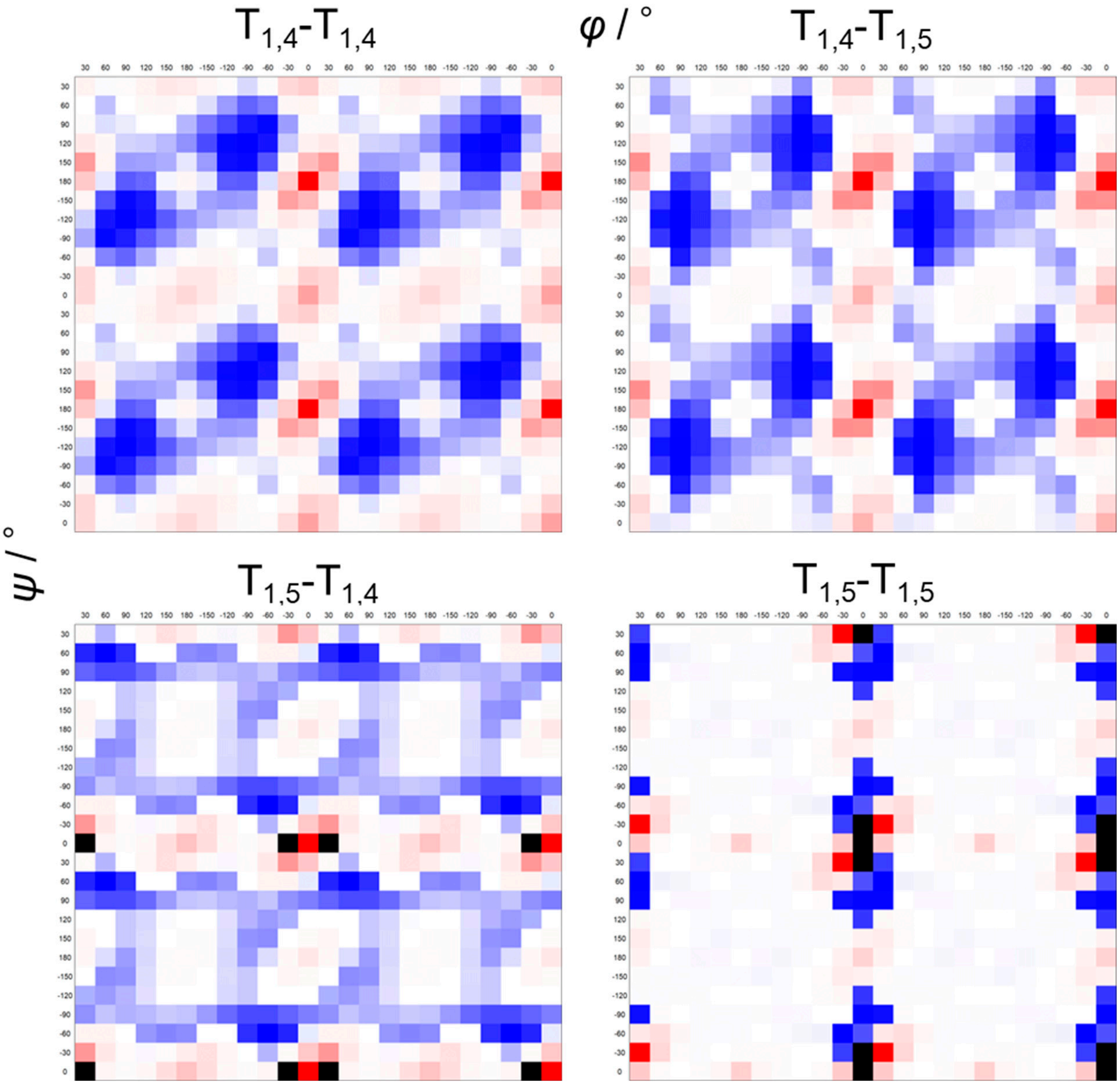

Figure S1. Formation energy map as a function of φ and ψ for $T_{1,4}$-$T_{1,4}$, $T_{1,4}$-$T_{1,5}$, $T_{1,5}$-$T_{1,4}$, and $T_{1,5}$-$T_{1,5}$. Note that red ((R,G,B) = (255,0,0)) is assigned to the data with the highest energy, white ((R,G,B) = (0,0,0)) to the median, blue ((R,G,B) = (0,0,255)) to the data with the lowest energy, and black to no data (calculation unsuccessful). in each diagram. Note: comparison of colors between different diagrams is not meaningful in this figure.

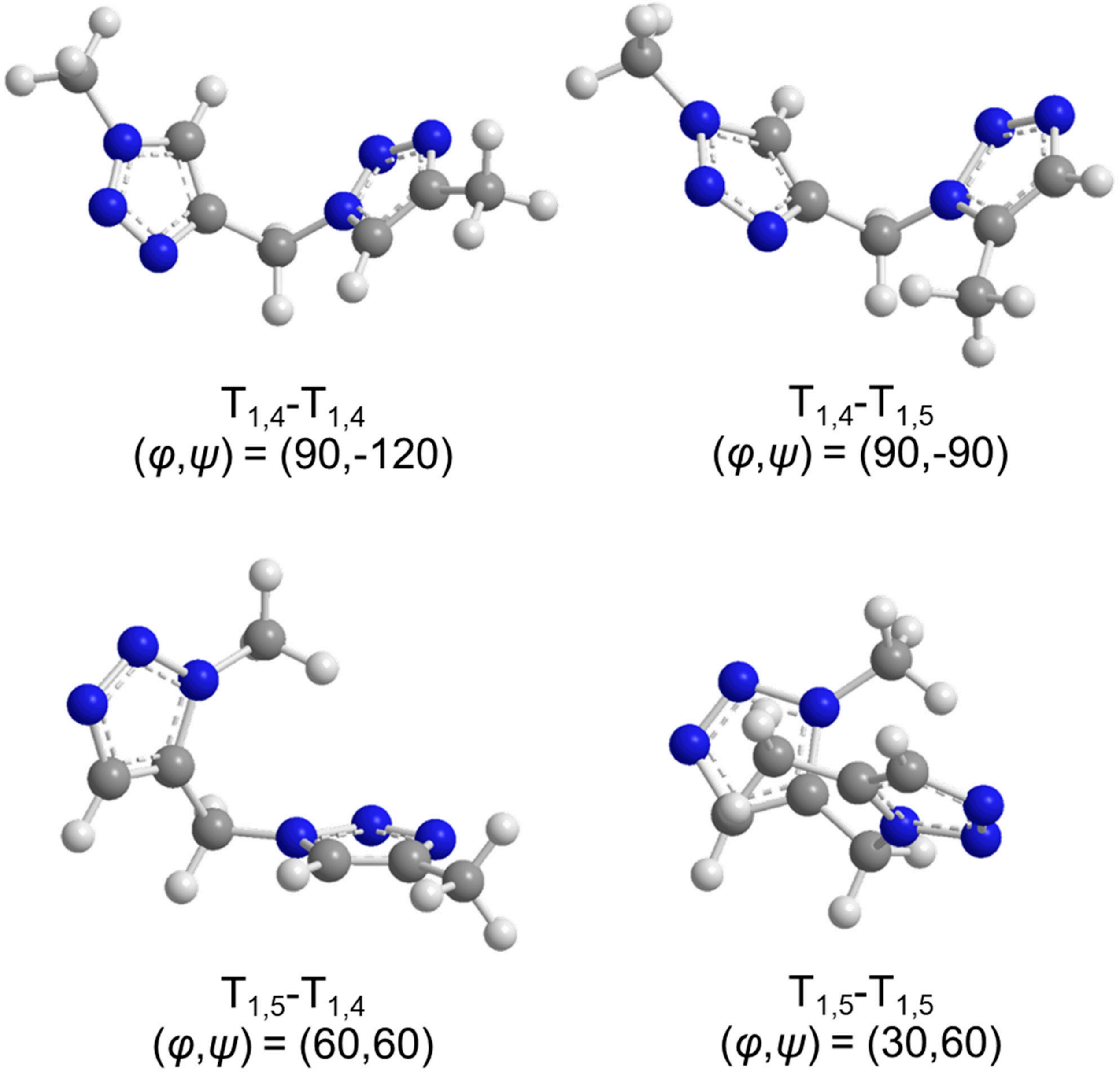

Figure S2. 3D structures for most stable conformers of $T_{1,4}$-$T_{1,4}$ with (90,−120), $T_{1,4}$-$T_{1,5}$ with (90,−90), $T_{1,5}$-$T_{1,4}$ with (60,60) and $T_{1,5}$-$T_{1,5}$ with (30,60).

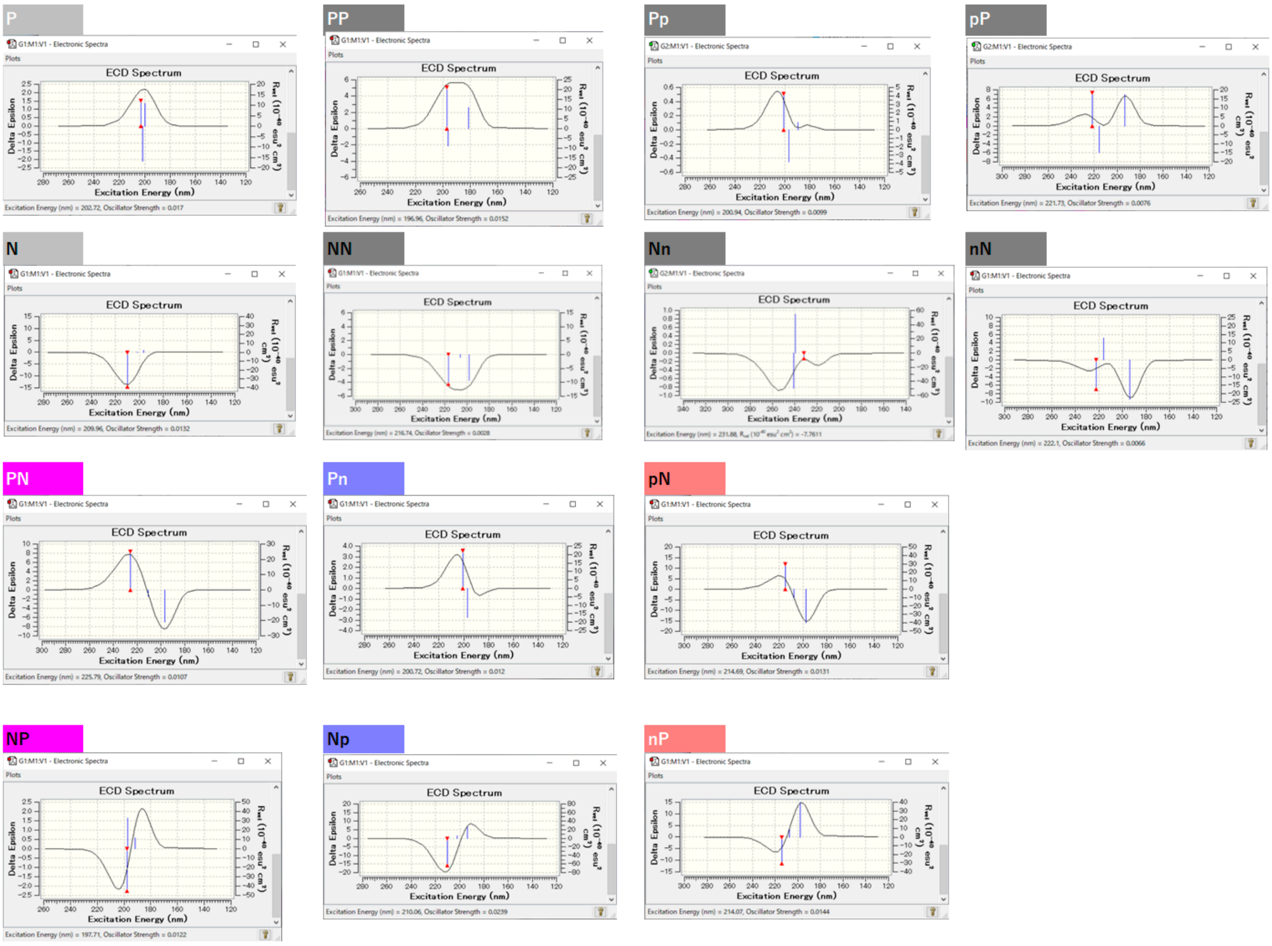

Figure S3. Classification of CD spectra.

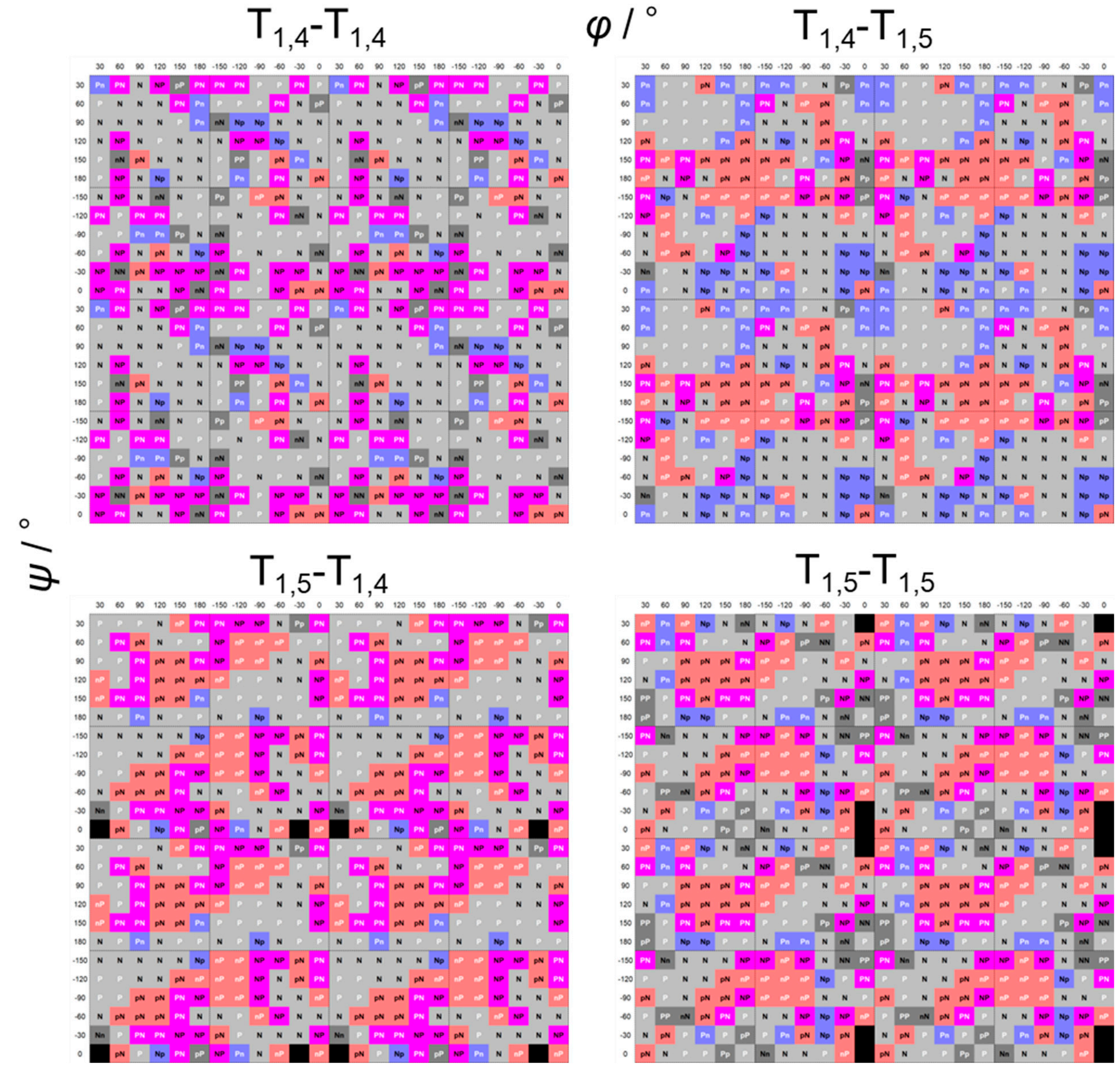

Figure S4. Classification of simulated CD spectra against φ and ψ for (a) $T_{1,4}$-$T_{1,4}$, (b) $T_{1,4}$-$T_{1,5}$, (c) $T_{1,5}$-$T_{1,4}$, and (d) $T_{1,5}$-$T_{1,5}$.

Table S5. Difference in formation energy ΔE and molar ratio N/N_0 for five pairs ((φ,ψ) and ($-\varphi,-\psi$)) for most energetically stable conformers of $T_{1,4}$-$T_{1,4}$.

Conformer	E / Hartree	ΔE / Hartree	ΔE / J mol^{-1}	N/N_0
$T_{1,4}$-$T_{1,4}$ (90,−120)	−601.11881430	0	0	1
$T_{1,4}$-$T_{1,4}$ (60,−90)	−601.11845790	0.00035640	935.73	0.687
$T_{1,4}$-$T_{1,4}$ (90,−150)	−601.11843160	0.00038270	1004.78	0.668
$T_{1,4}$-$T_{1,4}$ (120,−120)	−601.11816708	0.00064722	1699.28	0.506
$T_{1,4}$-$T_{1,4}$ (60,−120)	−601.11812720	0.00068710	1803.98	0.485
$T_{1,4}$-$T_{1,4}$ (−90,120)	−601.11887484	0	0	1.000
$T_{1,4}$-$T_{1,4}$ (−60,90)	−601.11849879	0.00037605	987.32	0.673
$T_{1,4}$-$T_{1,4}$ (−90,150)	−601.11839705	0.00047779	1254.44	0.605
$T_{1,4}$-$T_{1,4}$ (−120,120)	−601.11847058	0.00040426	1061.38	0.653
$T_{1,4}$-$T_{1,4}$ (−60,120)	−601.11813422	0.00074062	1944.50	0.459

Table S6. Difference in formation energy ΔE and molar ratio N/N_0 for five pairs ((φ,ψ) and ($-\varphi,-\psi$)) for most energetically stable conformers of $T_{1,4}$-$T_{1,5}$.

Conformer	E / Hartree	ΔE / Hartree	ΔE / J mol^{-1}	N/N_0
$T_{1,4}$-$T_{1,5}$ (90,−90)	−601.13742413	0	0	1
$T_{1,4}$-$T_{1,5}$ (90,−120)	−601.13735465	0.00006948	182.42	0.929
$T_{1,4}$-$T_{1,5}$ (90,−150)	−601.13712308	0.00030105	790.41	0.728
$T_{1,4}$-$T_{1,5}$ (60,−120)	−601.13663098	0.00079315	2082.42	0.434
$T_{1,4}$-$T_{1,5}$ (90,−60)	−601.13646254	0.00096159	2524.65	0.363
$T_{1,4}$-$T_{1,5}$ (−90,90)	−601.13742481	0	0	1.000
$T_{1,4}$-$T_{1,5}$ (−90,120)	−601.13735225	0.00007256	190.51	0.926
$T_{1,4}$-$T_{1,5}$ (−90,150)	−601.13712375	0.00030106	790.43	0.728
$T_{1,4}$-$T_{1,5}$ (−60,120)	−601.13662961	0.00079520	2087.80	0.433
$T_{1,4}$-$T_{1,5}$ (−90,60)	−601.13646044	0.00096437	2531.95	0.362

Table S7. Difference in formation energy Δ*E* and molar ratio N/N_0 for five pairs ((φ,ψ) and ($-\varphi$,$-\psi$)) for most energetically stable conformers of $T_{1,5}$-$T_{1,4}$.

Conformer	*E* / Hartree	Δ*E* / Hartree	Δ*E* / J mol^{-1}	N/N_0
$T_{1,5}$-$T_{1,4}$ (60,60)	−601.13755367	0	0	1
$T_{1,5}$-$T_{1,4}$ (30,60)	−601.13624727	0.00130640	3429.95	0.253
$T_{1,5}$-$T_{1,4}$ (90,60)	−601.13597379	0.00157988	4147.97	0.190
$T_{1,5}$-$T_{1,4}$ (60,90)	−601.13520073	0.00235294	6177.64	0.084
$T_{1,5}$-$T_{1,4}$ (90,90)	−601.13515012	0.00240355	6310.52	0.080
$T_{1,5}$-$T_{1,4}$ (−60,−60)	−601.13755417	0	0	1
$T_{1,5}$-$T_{1,4}$ (−30,−60)	−601.13624665	0.00130752	3432.89	0.252
$T_{1,5}$-$T_{1,4}$ (−90,−60)	−601.13596908	0.00158509	4161.65	0.189
$T_{1,5}$-$T_{1,4}$ (−60,−90)	−601.13519936	0.00235481	6182.55	0.084
$T_{1,5}$-$T_{1,4}$ (−90,−90)	−601.13514429	0.00240988	6327.14	0.079

Table S8. Difference in formation energy Δ*E* and molar ratio N/N_0 for five pairs ((φ,ψ) and ($-\varphi$,$-\psi$)) for most energetically stable conformers of $T_{1,5}$-$T_{1,5}$.

Conformer	*E* / Hartree	Δ*E* / Hartree	Δ*E* / J mol^{-1}	N/N_0
$T_{1,5}$-$T_{1,5}$ (30,60)	−601.13039652	0	0	1
$T_{1,5}$-$T_{1,5}$ (0,90)	−601.11958890	0.01080762	28375.41	0.000
$T_{1,5}$-$T_{1,5}$ (30,90)	−601.11935870	0.01103782	28979.80	0.000
$T_{1,5}$-$T_{1,5}$ (−30,90)	−601.11405186	0.01634466	42912.90	0.000
$T_{1,5}$-$T_{1,5}$ (0,120)	−601.08651303	0.04388349	115216.10	0.000
$T_{1,5}$-$T_{1,5}$ (−30,−60)	−601.13039752	0	0	1
$T_{1,5}$-$T_{1,5}$ (0,−90)	−601.11958807	0.01080945	28380.21	0.000
$T_{1,5}$-$T_{1,5}$ (−30,−90)	−600.93654890	0.19384862	508949.55	0.000
$T_{1,5}$-$T_{1,5}$ (30,−90)	−601.11402921	0.01636831	42975.00	0.000
$T_{1,5}$-$T_{1,5}$ (0,−120)	−601.08651334	0.04388418	115217.91	0.000
